# Candida Associated Bloodstream Infections in Pediatric Hematology Patients: A Single Center Experience

**DOI:** 10.4084/MJHID.2016.018

**Published:** 2016-03-01

**Authors:** Dilek Gurlek Gokcebay, Nese Yarali, Pamir Isik, Cengiz Bayram, Aslinur Ozkaya-Parlakay, Abdurrahman Kara, Bahattin Tunc

**Affiliations:** 1 Ankara Children’s Hematology and Oncology Hospital, Department of Pediatric Hematology, Ankara, Turkey; 2 Ankara Children’s Hematology and Oncology Hospital, Department of Pediatric Infectious Disease, Ankara, Turkey

## Abstract

**Background and Objectives:**

Candida-associated bloodstream infections are frequent and potentially life-threatening conditions in hematology patients. The aim of this study is to evaluate the characteristics, risk factors, and outcome of Candida-associated bloodstream infections in children with hematological diseases.

**Methods:**

The medical records of the patients with hematological diseases and hematopoietic stem cell transplantation (HSCT) recipients who were diagnosed as Candida-associated bloodstream infection between February 2010 and February 2014 were reviewed retrospectively.

**Results:**

Thirty episodes of candidemia involving 26 patients (38% female, and 62% male) with a median age of 7-year (range; 1 to 17) were noted. The incidence of candidemia in our study was 5.2 per 1000 hospital admissions. Infections with non-*albicans Candida* spp. occurred more frequently (63%) and *C. krusei* was the predominant microorganism among non-*albicans Candida* spp. (37%). *Candida albicans* was isolated from 11 of the 30 episodes (37%). Twenty-six of the episodes (88%) patients had a central venous catheter (CVC) prior to candidemia, and they were removed in 16 (62%). Thirty-day mortality rate was 20%. Isolated *Candida* spp, underlying disease and its status, presence of mucositis, neutropenia, using of broad spectrum antibiotics, corticosteroids or total parenteral nutrition were not identified as predictors of outcome. Multivariate analysis revealed that CVCs kept in place was the only significant factor associated with mortality (OR, 0.07; 95% CI, 0.006–0.716).

**Conclusions:**

Candida-associated bloodstream infections were common in children with hematological diseases and HSCT recipients, particularly in patients with CVCs. In addition to appropriate antifungal therapy, CVC removal improves the outcome of candidemia in children with hematological disease.

## Introduction

Candida-associated bloodstream infections have high morbidity and mortality in immune-compromised children like leukemia, aplastic anemia or undergoing hematopoietic stem cell transplantation (HSCT).^1^–^3^ Most of these infections are related to central venous catheter (CVC), and candidemia represents 10% of hospital-acquired CVC-related bloodstream infections.^4^,^5^ Furthermore, treatment with wide spectrum antibiotics, corticosteroids or chemotherapeutic drugs, invasive procedures, and prolonged neutropenia often predispose children to the fungal infections.^3^ In Turkey, nosocomial infection rate due to *Candida spp*. was reported as 3.22 per 1000 patient-days, and 42.9% of them were bloodstream infections.^6^ Celebi et al. also reported that 4.5% of the catheter related bloodstream infections were due to *C. albicans* in children with malignant disease.^7^

*Candida species (spp.)* commonly present in the gastrointestinal tract, may produce invasive infections in the immune compromised host when mucosal barrier is disrupted, or normal gastrointestinal flora is abrogated by antimicrobial treatment.^5^ *Candida spp*. may also colonize to form biofilm formation at CVCs that results in compromised antifungal treatment and cause recurrence of fungal infections after cessation of antifungal therapy **[**3**]**. The Infectious Diseases Society of America (IDSA), Fourth European Conference on Infections on Leukemia (ECIL-4), and European Society of Clinical Microbiology and Infectious Diseases guidelines (ESCMID) recommend systemic antifungal therapy and CVC removal for bloodstream infections associated with Candida.^8^,^9^,^10^ Even though many new antifungal drugs have been discovered, the candidiasis-related mortality rate remains high, ranging from 7.7% to 26%.^11^

The aim of our study is to evaluate the characteristics, risk factors, and outcome of Candida-associated bloodstream infections in children with hematological diseases.

## Material and Methods

This retrospective study included children with a hematological disease such as leukemia, severe aplastic anemia and HSCT recipients who had a persistent fever and were diagnosed as “Candida-associated bloodstream infection-candidemia” at the Hematology Department of Ankara Children’s Hematology and Oncology Hospital between February 2010 and February 2014. Ankara Children’s Hematology and Oncology Hospital is a large tertiary pediatric hospital with hematology-oncology research and treatment center, and has 35 hematology and 10 HSCT beds, and it has around 1450 hematology inpatient admissions per-year. Eligible children were reviewed from their electronic medical records and microbiology laboratory reports for blood and CVC cultures, which yielded *Candida spp*. Demographic and clinical data of the patients including age, gender, type and stage of primary disease, presence and type of CVC, time from the end of last chemotherapy to the positive culture, presence and duration of neutropenia, history of HSCT and graft-versus-host disease (GvHD), presence of mucositis, administration of total parenteral nutrition (TPN), use of broad spectrum antibiotics, administration of corticosteroids or cyclosporine within 14 days of positive blood culture were recorded.

As a policy, in our department patients with relapsed acute myeloblastic leukemia (AML) and acquired aplastic anemia receive fluconazole prophylaxis during relapse protocol and immunosuppressive therapy. Hematopoietic SCT recipients receive antifungal prophylaxis with fluconazole for 30 days after transplantation. Patients with febrile neutropenia first receive empiric intravenous extended spectrum penicillin with an aminoglycoside, and if fever persists for 3–5 days glycopeptides and empiric/preemptive antifungal therapy is added. Liposomal amphotericin B is the most common antifungal drug that is used empirically; caspofungin or voriconazole are the other antifungal drugs which are used for either suspected or proven fungal infections.

Blood specimens were cultured in BACTEC Blood Culture System (Becton Dickinson Diagnostic Instrument Systems, Towson, MD, USA). Candidemia was defined as positivity of blood culture obtained from either peripheral vein and/or CVC, associated with clinical symptoms of bloodstream infection such as fever or hypotension. All positive CVC cultures were also assessed for the interval between the isolation of *Candida spp*. and CVC removal.

Mortality attributable to candidemia was defined as death within 30 days after the first positive blood or catheter culture in the absence of any apparent cause for death.

### Statistics

Analysis of data was primarily descriptive, using standard deviations, ranges, and mean and median values. Categorical variables were analyzed using the chi-square test. After doing univariate analysis, all variables reaching a 95% level of significance were included in a binary logistic regression analysis. Survival rates were calculated by Kaplan-Meier analysis and the log rank test. All analyses were performed using SPSS 18 for Windows (SPSS Inc., Chicago, IL, USA). p≤ 0.05 was considered statistically significant.

## Results

Thirty episodes of candidemia involving 26 patients (38% female, and 62% male) were detected with a median age of 7-year (range; 1 to 17 years). Recurrent episodes of candidemia occurred in two patients with leukemia, one patient with severe acquired aplastic anemia, and one with severe combined immunodeficiency disease (SCID) undergoing HSCT. The incidence of candidemia was 5.2 per 1000 hospital admissions. The demographic characteristics of the patients with candidemia were shown in Table 1. Acute leukemia was the most common underlying disease (17 patients; 65%), and 6 (35%) of these leukemic patients were at induction phase of chemotherapy, and another 6 (35%) patients were at relapse treatment. Nine patients developed candidemia after allogeneic HSCT, and four patients had severe acquired aplastic anemia. During the study period, the overall incidence of candidemia episodes in patients with ALL, AML, AA, and HSCT were 5.4%, 3%, 30%, and 6.9%, respectively.

Risk factors associated with outcome were presented in Table 2. Eighty three percent of the patients had received broad-spectrum antibiotics with a median of 14 days (range, 1–180 days), and 83% of the patients had received chemotherapy one month prior to candidemia. Candidemia occurred during severe neutropenia in 73% of patients, lasting a median of 21 days (range, 7–110 days). Furthermore, 47%, 30% and 36% of the patients developed candidemia during the corticosteroid, total parenteral nutrition, and cyclosporine treatment, respectively. In 12 of the episodes (40%), patients had a history of fluconazole prophylaxis.

Isolated *Candida* species were as follows: *C. albicans* (n=11), *C. krusei* (n=7), *C. parapsilosis* (n=2), *C. tropicalis* (n=2), *C. cifferii* (n=1), *C. glabrata* (n=1), *C. dubliensis* (n=1), *C. lusitanae* (n=1), *C. kefyr* (n=1), and non-*albicans Candida* that could not be classified (n=3). Distribution of isolated *Candida spp*. and the isolation sites were shown in Table 3.

Twenty-three of the 26 patients (88%) had CVC prior to candidemia. Hickman double lumen catheters had been inserted in six out of nine patients before HSCT, and nontunnelled jugular catheters had been inserted in another three patients. Patients with leukemia had port catheters. Central VCs were removed in 16 of the 26 episodes (62%) and were remained in place in 11, because of patient’s poor clinical condition or coagulopathy. The median duration between the isolation of *Candida spp*. to CVC removal was 7 days (range, 2–22 days). Only one of the CVC tip cultures was positive, and *C. krusei* was isolated. Twelve patients (40%) were on fluconazole prophylaxis, and nine of them (75%) had non-*albicans Candida* infection (Table 4). We found no association between fluconazole prophylaxis and isolated *Candida* spp. We also found no difference between isolated *Candida spp*. *(albicans* or non-*albicans)* in children who underwent HSCT.

**Figure 1:**
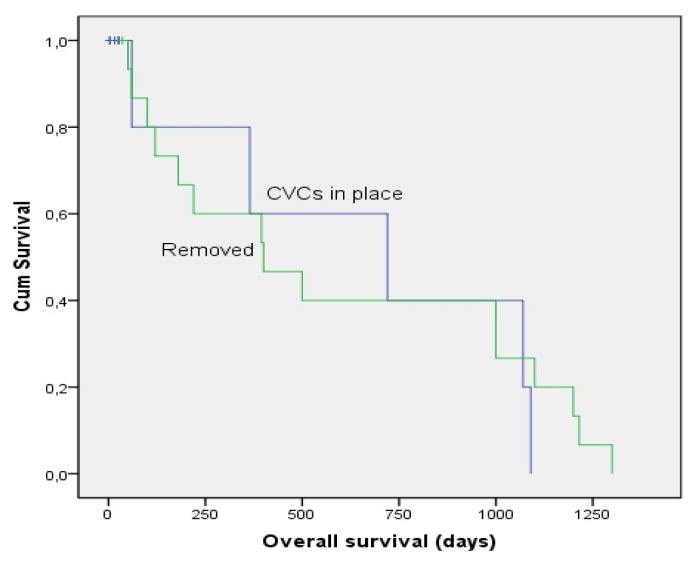
Kaplan-Meier estimates of overall survival of patients with CVCs kept in place and removed.

Overall 8 of the 26 patients (27%) died with Candida-related septicemia in 20 days (range, 1–34 days) from the positive culture. All patients who died from candidemia had CVC except one. In univariate analysis, underlying disease and is status (remission or non-remission), the presence of mucositis, presence of neutropenia, using of wide spectrum antibiotics, corticosteroids, cyclosporine, TPN or isolated *Candida spp*. were not identified as significant predictors of outcome. However, fluconazole prophylaxis before candidemia and removal of CVC were important factors for a better outcome (p=0.034, and p=0.012, respectively, Table 2). Multivariate analysis showed only CVCs kept in place was associated with mortality (p=0.03; OR, 0.07; 95% CI, 0.006–0.716). Kaplan Meier analysis revealed that the three months estimated survival of patients with CVCs kept in place was 80% [SE 0.179], whereas this figure was 86.6% [SE 0.088] with CVC removal (p=0.69) (Figure 1). All surviving patients had a last negative culture following treatment.

## Discussion

Candida infections is an important cause of morbidity in critically ill children. However, studies on clinical and epidemiological features of Candida-associated bloodstream infections in immune compromised children with hematological diseases are scarce. In this study, the incidence of candidemia was 5.2 per 1000 hospital admissions in immune-compromised children with hematologic diseases, which was lower than the Brasilian study^12^ which reported 19.14 episodes per 1000 admission. Our study also revealed that non-*albicans Candida* spp. occurred more frequently (63%) and *C. krusei* was the predominant microorganism among non-*albicans Candida spp*. (37%). It confirms the previous reports, which show the increasing incidence of non-*albicans* Candida infections in patients with cancer and hematological malignancies.^12^–^17^ The most prominent species that have been reported were *C. parapsilosis* in patients with CVC-related infections, but non-*albicans Candida* isolates, *C*. *glabrata, tropicalis* and *krusei* in patients without CVC.^14^ Several risk factors have been reported to be associated with non-*albicans* Candida infections including azole prophylaxis, neutropenia, type of the disease (leukemia) and HSCT.^13^,^18^ In the current study, we could not identify any specific risk factor for non-*albicans Candida* infection. Although administration of fluconazole prophylaxis might have resulted in the occurrence of more prevalent *C. krusei* infection which is inherently resistant to fluconazole therapy; but we found no relation between fluconazole prophylaxis and isolated *Candida spp*. in this study.

Zaoutis et al. reported that the presence of a CVC, a diagnosis of malignancy and receipt of antimicrobials with activity against anaerobic bacteria for >3 days were independently associated with the development of candidemia.^3^ History of using broad-spectrum antibiotics, prior intensive chemotherapy and neutropenia were also observed frequently in our patients with candidemia.

Indwelling CVC has been previously identified to be a risk factor for candidemia in patients with malignancy.^4^,^12^,^19^ The guidelines for the treatment of candidemia recommend the removal of CVC in the presence of bloodstream infections.^8^,^9^,^10^ However, management of infected CVC in neutropenic patients is still controversial, because of patient’s poor clinical condition and potential surgical complications associated with CVC replacement. Most of our patients (88%) had CVC prior to candidemia, and CVC could not have been removed in 38% of the episodes due to patient’s condition. Candidemia is an independent risk factor for predicting death in patients with nosocomial bloodstream infections.^20^ The candidemia-associated mortality rate has been reported as 20–24%.^13^,^16^ In the current study, the overall mortality rate of candidemia was 27%. The mortality rate was 54% in patients with CVCs kept in place, and it was 6% in children whom CVC had been removed. The only factor that was significantly associated with mortality was CVC removal. However, removal of CVC did not influence patient’s overall survival. Removing the CVC in a patient with a poor condition or with severe coagulopathy such as disseminated intravascular coagulation or platelet refractoriness, may prove to be difficult. Nevertheless, we recommend CVC removal as soon as the patient’s condition permits.

## Conclusion

The current study was limited by its retrospective design and low sample size in a single center experience. We conclude that as long as the medical condition permits, CVC removal should be considered in patients with hematological diseases and Candida*-*associated bloodstream infections. Further large-scale studies are warranted to evaluate the contribution of the other risk factors, such as pre-existent medical conditions, to outcome in children with candidemia.

## Figures and Tables

**Table 1:** Clinical characteristics of the patients with candidemia.

	*n: 26*	*%*
***Gender***		
Female	10	38
Male	16	62
***Age (year)*** (median, range)	7 (1–17)	
***Underlying disease***		
ALL	12	46
AML	2	8
Aplastic anemia	3	11
HSCT:	9	35
• Leukemia	3	11
• SCID	2	8
• Thalassemia major	2	8
• Aplastic anemia	1	4
• Infantile Crohn disease	1	4

ALL: Acute lymphoblastic leukemia; AML: Acute myeloblastic leukemia; SCID: Severe combined immunodeficiency disease.

**Table 2:** Risk factors associated with outcome

*Factors*		*Number of episodes (n=30) (%)*	*Number of died patients (n=8) (%)*	*p*†
**Underlying disease**	benign	10 (33)	5 (63)	0.07
	malign	20 (77)	3 (38)	
**Disease Status**	remission	13 (43)	1 (13)	1
	non-remission	7 (23)	2 (25)	
**Presence of oral mucositis**		11 (37)	5 (63)	0.1
**Use of broad spectrum antibiotics**		25 (83)	8 (100)	0.28
**Severe neutropenia** (<500/mm^3^)		22 (73)	8 (100)	0.07
**Prior chemotherapy** *		25 (83)	6 (75)	0.06
**Total parenteral nutrition**		9 (30)	5 (63)	0.055
**Presence of CVC**		26 (88)	6 (75)	0.28
**Removal of CVC** (n=26)		16 (62)	1 (13)	**0.012**
**Use of corticosteroids**		14 (47)	3 (38)	0.68
**Use of cyclosporine**		11 (36)	5 (63)	0.1
**Fluconazole prophylaxis**		12 (40)	6 (75)	**0.034**

* One month before positive culture

† Data were analysed using chi-square test, and p≤ 0.05 was considered statistically significant.

CVC: Central venous catheter.

**Table 3:** Distribution of isolated *Candida* species and source of culture.

*Candida* species	*n*	*%*
*C. albicans*	11	37
*C. krusei*	7	24
*C. parapsilosis*	2	7
*C. tropicalis*	2	7
*C. cifferii*	1	3
*C. glabrata*	1	3
*C. dubliensis*	1	3
*C. lusitanea*	1	3
*C. kefyr*	1	3
*Candida spp*.	3	10
**Source of blood culture**		
Peripheral vein	12	40
Port catheter	2	7
CVC	7	24
Peripheral vein and port catheter	8	26
Peripheral vein and CVC	1	3

CVC: Central venous catheter.

**Table 4:** Characteristics and outcome of the patients

Patient	Gender	Age	Disease	Fluconazole prophylaxis	Candida spp.	CVC removal	Outcome
**1**	M	17	AA	yes	*C. cifferii*	no	died (10)
**2**	F	9	AA*	yes	*C. krusei*	yes (4)	survived
**3**	F	16	AML*	yes	*C. glabrata*	no	died (4)
**4**	M	4	ALL*	yes	*C. lusitanea*	yes (3)	survived
**5**	M	3	ALL	no	*C. kefyr*	yes (1)	survived
**6**	M	14	AML	yes	*C. dubliensis*	no	survived
**7**	M	1	TM*	yes	*C. parapsilosis*	yes (2)	survived
**8**	F	13	TM*	yes	*C. albicans*	no	died (16)
**9**	F	1	SCID*	yes	*Candida spp.*	no	died (32)
**10**	F	4	ALL	no	*C. krusei*	no	survived
**11**	F	2	SCID*	yes	*C. albicans*	yes (7)	died (36)
**12**	M	5	ALL	no	*C. tropicalis*	yes (10)	survived
	2.episode			no	*C. albicans*	no	died (3)
**13**	M	7	ALL	no	*C. albicans*	yes (22)	survived
**14**	F	9	ALL	no	*C. parapsilosis*	yes (16)	survived
**15**	F	16	AA	no	*C. krusei*	yes (15)	survived
	2.episode			no	*C. krusei*	no	survived
**16**	M	2	CD*	yes	*C. albicans*	no	died (27)
**17**	M	16	ALL*	yes	*C. tropicalis*	yes (9)	survived
**18**	M	12	AA	yes	*Candida spp.*	no	survived
**19**	M	17	ALL	no	*C. krusei*	yes (20)	survived
**20**	F	7	AML	no	*C. krusei*	no	died (25)
**21**	M	11	ALL	no	*Candida spp.*	no	survived
**22**	M	2	ALL	no	*C. albicans*	no	survived
	2.episode	2		no	*C. albicans*	yes (7)	survived
**23**	F	7	ALL	no	*C. albicans*	yes (7)	survived
**24**	M	1	ALL	no	*C. albicans*	no	survived
**25**	M	3	ALL	no	*Candida spp.*	yes (4)	survived
	2.episode			no	*C. albicans*	yes (6)	survived
**26**	M	17	ALL	no	*C. krusei*	yes (21)	survived

* HSCT patients.

() refers time (days) after the onset of candidemia.

AA: Aplastic anemia; ALL: Acute lymphoblastic leukemia; AML: Acute myeloblastic leukemia; CD: Infantile Crohn disease; TM: Thalassemia major; SCID: Severe combined immunodeficiency disease.
